# Current Trends in Water-in-Diesel Emulsion as a Fuel

**DOI:** 10.1155/2014/527472

**Published:** 2014-01-20

**Authors:** Mohammed Yahaya Khan, Z. A. Abdul Karim, Ftwi Yohaness Hagos, A. Rashid A. Aziz, Isa M. Tan

**Affiliations:** ^1^Department of Mechanical Engineering, Universiti Teknologi Petronas, Seri Iskandar, 31750 Tronoh, Perak, Malaysia; ^2^Department of Fundamental & Applied Science, Universiti Teknologi Petronas, Seri Iskandar, 31750 Tronoh, Perak, Malaysia

## Abstract

Water-in-diesel emulsion (WiDE) is an alternative fuel for CI engines that can be employed with the existing engine setup with no additional engine retrofitting. It has benefits of simultaneous reduction of both NO_*x*_ and particulate matters in addition to its impact in the combustion efficiency improvement, although this needs further investigation. This review paper addresses the type of emulsion, the microexplosion phenomenon, emulsion stability and physiochemical improvement, and effect of water content on the combustion and emissions of WiDE fuel. The review also covers the recent experimental methodologies used in the investigation of WiDE for both transport and stationary engine applications. In this review, the fuel injection pump and spray nozzle arrangement has been found to be the most critical components as far as the secondary atomization is concerned and further investigation of the effect of these components in the microexplosion of the emulsion is suggested to be center of focus.

## 1. Introduction

Diesel engines offer better fuel to power conversion efficiency and due to their better fuel economy, diesel engines are the dominant class of engines in mass transportation, heavy industries, and agricultural sectors. In spite of their preferable advantages, they are one of the major pollution contributors to the environment. Primary pollutants emitted from diesel engines are particulate matters (PM), black smoke, nitrogen oxides (NO_*x*_), sulphur oxides (SO_*x*_), unburned hydrocarbon (HC), carbon monoxide (CO), and carbon dioxide (CO_2_) [1]. Increasing stringent regulation on exhaust emissions drives a major research endeavour in engine development in order to reduce these pollutants [2, 3]. Significant reduction targets include reduction of PM from 0.025 g/km in Euro 4 (2005) to 0.0045 g/km in Euro 6 (2014) for both CI passenger cars and light commercial vehicles, which account for a 82% reduction. Similar reduction targets are also imposed on heavy-duty engines with a reduction of 50% in PM emission [4].

Modern hardware-based solutions for pollution control such as diesel particulate filters (DPF), high-pressure fuel injection equipment (FIE), and sophisticated piezoinjectors and associated control systems are avenues being followed by engine designers and manufacturers. However, these technologies come with high price tags and cannot be fitted to existing engines. Therefore, there is a pressing need for appropriate technology that can be applied to these existing engines. One such possibility is to develop fuel-based solutions which do not rely on new hardware to control the combustion process and hence the emissions. Research showed that WiDE used as an alternative fuel in CI engines can lead to reductions in the adiabatic flame temperature resulting in measurable reductions in the NO_*x*_ emissions [5–8]. There are many advantages to using emulsion fuels, such as more complete combustion, leading to better fuel economy, and cleaner burning fuels with fewer emissions.

The main mechanism causing the reduction in NO_*x*_ emissions seems to be the decrease in the temperature of the combustion products as a result of vaporisation of the liquid water and consequent dilution of the gas phase species. As for PM emissions, the presence of water during the intensive formation of soot particles seems to reduce the rate of formation of soot particles and enhance their burnout by increased concentration of oxidation species such as OH [9].

Water can be introduced into the combustion chamber in different ways as follows: (a) introduction of water with the inlet air in liquid or vapour form, (b) parallel water and diesel injections, and (c) WiDE with or without surfactants. While the first two methods of water introduction are subjected to additional cost of water injection system and engine corrosion problems [10], the later method has been regarded as the most effective technique for the simultaneous reduction of both particulate matters and NO_*x*_ [11]. Moreover, WiDE is a convenient renewable fuel option as the existing engine does not require any prior or postmodification.

Till date, research on WiDE is active and even its comparative advantage to its base petroleum fuels is not precisely known. The reasons behind this is the lack of understanding of the combustion phenomenon associated with the formation of soot inside the combustion chamber, complexity in nature of combustion chamber, unknown end-to-end physical path of emulsion (evaporation and mixing), and the effect of microexplosion phenomenon inside the combustion chamber. Because of these reasons, the reported result of researches in this area has been inconsistent [9] as far as the brake thermal efficiency, brake specific fuel consumption, and pollutant formation are concerned. In addition to the above mentioned problems, there are many factors affecting the atomization and the general combustion process on top of the wide operating variables. The researches were mainly focused in specific engine operation variables and due to this, the results has become very difficult to draw a general conclusion. The results reported by different researchers are often conflicting, sometimes generating results that are even worse than pure diesel fuel [12]. As a result, there is still a need for further investigation especially with fuelling WiDE into compression ignition engine by varying the applied conditions. This paper will review the current status of water-in-diesel emulsions so that to bring research works in this area under one document and to further enlighten the possible area of intervention for researchers.

## 2. Methodologies Used in Water-in-Diesel Emulsion Study

Different methodologies have been used to study WiDE as a fuel for internal combustion engine both inside and outside the engine combustion chamber. Abu-Zaid [13] has used horizontal stainless steel and aluminum surfaces to study the evaporation of water-in-diesel and water-in-kerosene emulsion by varying the surface temperature from 100 to 460°C at atmospheric pressure. The evaporation characteristics of the droplet, effect of water concentration, and total evaporation time were investigated experimentally. Tanaka et al. [14, 15] and Tsue et al. [16] used the same horizontal hot surface to study the microexplosion of evaporating droplet. While the hot surface used by Abu-Zaid [13] was exposed to the atmosphere, the apparatus used by Tanaka and coworkers [14, 15] and Tsue and coworkers [16] was made of duralumin and the hot surface was isolated from the atmosphere with a high pressure cylindrical chamber. The major objectives of the experimental investigations made by this apparatus were to study the effect of ambient pressure on the start of microexplosion [15], the effect of the water concentration, base fuel property, and surface temperature on the statistical characteristics of the start of microexplosion [14], and to study the start of microexplosion of the emulsion fuel droplet by the use of statistical analysis [16]. Watanabe et al. have used single droplet experiment where the emulsified droplet was suspended on a fine wire to study the breakup characteristics of a secondary atomization of an emulsion [17], Jeong et al. [18] studied the autoignition and microexplosion behavior of a single droplet, Jeong and Lee [19] investigated the autoignition and microexplosion behaviors of one-dimensional arrays of fuel droplets, and Morozumi and Saito [20] examined the microexplosion characteristics of the emulsion droplet. Yatsufusa et al. [21] have used air-assisted fuel atomizing burner to study the combustion and emission characteristics of WiDE.

The application of both hot surface and suspension single droplet as a means to examine both the evaporation and microexplosion phenomena is very important to predict the air-fuel mixing process and further the combustion and emission formation process. The results might not be accurate as the apparatuses are constructed with major assumptions. However, experimentation of evaporation and microexplosion phenomena and investigation of the effect of these phenomena in the combustion and emission formation inside the combustion chamber are extremely difficult tasks.

There have been other practices and methodologies used in the study of WiDE other than the above mentioned ones, such as WiDE and other test fuels in a diesel like constant volume combustion chamber and rapid compression expansion machine with controllable temperatures and pressures in the range of 293–923 K and 0.1–5.0 MPa, respectively [12, 20–26]. The effect of temperature and pressure on the microexplosion phenomenon was experimentally investigated by using this apparatus with the help of a multipulsed ruby laser holocamera with an off-axis image-plane optical path and a high speed camera [12]. The effects of injection pressure and water concentration on the spray combustion characteristics, like ignition delay and lift-off length of combustion of the emulsion, were investigated with the help of a diesel-like constant volume chamber. The high pressure and high temperatures were created by combustion of carbon-monoxide mixed with compressed air and oxygen and ignited by a spark plug [27]. Water-in-diesel microemulsion, WiDE, and conventional diesel fuels were experimentally investigated for their physical properties, spray behavior, and combustion characteristics. Spray cone angle, liquid phase penetration, droplet penetration, and vapor penetration were studied prior to ignition inside a controlled atmosphere similar to engine combustion chamber in a high pressure high temperature test rig. In this study, the size of water droplets in the WiDE and water-in-diesel microemulsion was further investigated with the help of a Nuclear Magnetic Resonance (NMR) diffusometry. Physical characterization of the test fuels was also achieved with controlled stress rheometer for rheology, sigma 70 tensiometer for surface tension, and Mettler Toledo DA-100 M density meter for density measurements [22]. The effect of water concentration and injection timing on the combustion performance and emission of an emulsified fuel were experimentally investigated in an engine like rapid compression and expansion machine, or RCEM. Successive flame image by high speed camera, pressure data, needle lift, and crank angle were taken in every 0.1° crank angle intervals [26].

Different types of four stroke engines with and without an optical access have been used to study the effect of WiDE on combustion and emissions [7, 28–35]. All researchers used similar arrangement in this type of experimental investigation except for the variation of size and technology of the components. The setup consisted of four stroke engine connected to eddy current dynamometer, high pressure- transducer for engine cylinder indicating to be instrumented in one cylinder, fuel flow rate meter, thermocouples for engine inlet and exhaust emissions, air inlet flow rate measurement, and exhaust gas analyzer for the measurement of NO_*x*_, CO, CO_2_, HC, and O_2_. Study of the combustion process was difficult since there has not been any optical access to the combustion chamber. As a result, more focus was given to the cylinder and thermocouple readings for the engine wall and inlet and exhaust temperatures to study the performance of combustion.

There are also significant researches of WiDE for CI engine application by numerical and mathematical modeling. These studies can be broadly categorized into modeling water-in-diesel spray with a focus on the microexplosion, droplet break-up and autoignition [12, 36–40]; heat release and engine performance modeling of combustion [41, 42] and emission formation modeling [43, 44].

## 3. Principle of Water in Diesel Emulsion

An emulsion is a mixture of two or more liquids immiscible in nature, one present as droplet, or dispersed phase distributed throughout the other, or the continuous phase [54]. It is generated by means of a mechanical agitation in the presence of surface active agents, sometimes called emulsifiers or surfactants, for stability. The surfactants possess a polar, or hydrophilic head and a nonpolar, or hydrophobic tail [55]. It is incorporated to weaken the surface tension of the medium in which it dissolves. When it is placed in an oil-water mixture, the polar groups orient towards the water and the nonpolar group orients towards the oil as it lowers the interfacial tension between the oil and water phases [56]. They are classified into cationic, anionic, amphoteric, and nonionic based on the type of polar group on the surfactant. For a best formation of appropriate surfactant, hydrophilic-lipophilic balance, or HLB (water liking-oil liking) score is developed. Low HLB tends to make water-in-oil-emulsion while those with a high HLB are more hydrophilic and tend to make oil-in-water-emulsion. The value of HLB ranges from 1 to 20.

The fact that emulsion is used as a fuel in diesel engine, it is recommended that it should be stable and this can be realized with the help of suitable surfactants. Surfactants should easily burn with no soot and free of sulfur and nitrogen [36]. Furthermore, they should have no impact on the physiochemical properties of the fuel. Usually the amount introduced in the emulsion process is in the range of 0.5–5% by volume ratio. The most common surfactants used in the water-in-diesel emulsion are sorbitan monooleate [9, 17, 30, 32] and polyethylene glycol sorbitan monooleate mixture [13, 24, 57, 58], polyethylene glycol sorbitan monooleate (polysorbate 80) and sorbitol sesquioleate (SSO) mixture [22], sorbitan monolaurate [27], gemini [32], polyoxyethylene nonylphenyl ether [15, 59, 60] solgen 40 and noigen TDS-30 (dai-ichi kogyo seiyaku) [16], polysorbate 20 (commercially known as tween 20) [33], detergent/liquid soap [21, 61], and t-octylphenoxy polyethoxy ethanol.

There have been a limited literature about the effect of surfactant on the characteristics of water-in-diesel emulsion as far as combustion and emissions are concerned [5]. Nadeem and coworkers have studied water-in-diesel emulsion with conventional (sorbitan monooleate) and gemini surfactants for main pollutant emissions by fuelling it in a four stroke and four cylinder engine test bed and concluded that for 15% water content, there is 71% reduction in PM emission with gemini surfactant water in diesel emulsion fuel [32].

There are two types of emulsification techniques, namely, two-phase (sometimes called primary) and three-phase emulsion (sometimes called multiphase or secondary emulsion to include complex emulsions with more than three liquid ingredients). The two-phase emulsion constitutes one continuous phase and one-dispersed phase liquids while the three-phase emulsion constitutes one continuous phase and two or more dispersed phase liquids. Although this paper is mainly focused at the water-in-diesel emulsion which is categorized under the two-phase emulsion, addressing the three-phase emulsion research, specifically those which are compared with two-phase emulsions is essential. Therefore, research work in the three-phase emulsion techniques has also been reviewed.

### 3.1. Three-Phase Emulsion

**Figure 1:**
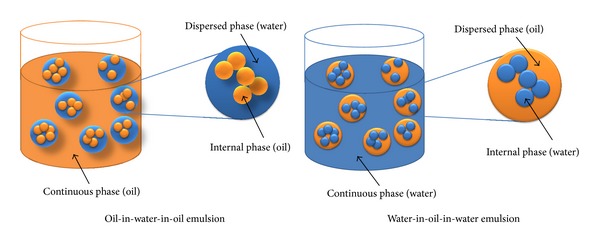
Concept of three-phase oil-in-water-in-oil and water-in-oil-in-water emulsions.

Two types of three-phase emulsions can be resulted from the three-phase emulsification technique (Figure 1) depending on the inner and outer phases, namely, oil-in-water-in-oil and water-in-oil-in-water emulsions. oil-in-water-in-oil emulsions are applicable for fueling purposes in the internal combustion engine while water-in-oil-in-water emulsion is applied in cosmetics, food, or pharmaceutical manufacturing [1]. A limited literature is available on the oil-in-water-in-oil emulsions application as a fuel in internal combustion engines. Three-phase emulsion can be prepared by three techniques, namely, phase inversion, mechanical agitation, and two-stage emulsion [1]. A two stage emulsification technique has been used for the preparation of three-phase oil-in-water-in-oil emulsion by many researchers [30, 31, 42, 43]. This technique, which is the most common technique in three-phase emulsion, uses both lipophilic and hydrophilic type of surfactants. First, a two-phase oil-in-water emulsion is prepared by using a hydrophilic type surfactant and a mechanical homogenizer machine. A lipophilic type of surfactant is then used to further emulsify the two-phase oil-in-water emulsion in oil and form three-phase oil-in-water-in-oil emulsion.

On study of emulsification characteristics property of three-phase oil-in-water-in-oil, Lin and Wang [1] have investigated the effect of homogenizing machine speed, oil/water ratio, HLB, and surfactant amount on the diameter of the liquid droplets, viscosity, and general stability of the three-phase emulsion. Based on their conclusion, there was a decrease in the diameter from 6 *μ*m to 2-3 *μ*m of the liquid droplets with an increase in the stirring speed of 2500 rpm to 7500 rpm. The viscosity of the three-phase oil-in-water-in-oil emulsion was also highly influenced by the oil/water ratio in which viscosity increased with an increase in water content in the inner phase. Furthermore, more stable three-phase emulsion was reported with a surfactant volume of 2% and HLB value in the range of 6–8 [1]. Another study by the same authors [31] was conducted to determine the effect of HLB value, water content, oil/water ratio, stir speed, and engine operating conditions on the performance and emissions of four-cylinder four stroke marine diesel engine. They have compared the two-phase and three-phase emulsion and the general emulsions with the base diesel fuel based on the engine performance and emission parameters. They have reported that the three-phase emulsion has lower brake specific fuel consumption, CO, NO_*x*_, and equivalence ratio but higher exhaust gas temperature compared to the two-phase emulsion. Moreover, the emulsion registered a lower exhaust gas temperature, O_2_, NO_*x*_, and smoke opacity, and higher CO_2_, CO, and equivalence ratio compared to the base diesel fuel. Similar research by Lin and Chen [30] was conducted to investigate the effect of emulsifying mechanism on the performance and emission of two-phase and three-phase emulsion fueled in four-cylinder four stroke marine diesel engine. In this study, they have compared both two-phase water-in-oil emulsion and three-phase oil-in-water-in-oil emulsions prepared with ultrasonic vibrator and mechanical homogenizer. The same authors, on a separate publication, have also investigated the effect of the time of emulsification, quantity, and HLB of surfactant on the emulsified fuel properties of two-phase water-in-oil and oil-in-water emulsions and three-phase oil-in-water-in-oil emulsion prepared by ultrasonic vibrator. They have compared oil-in-water and water-in-oil two-phase emulsions with respect to their temperature rise with increase in emulsification time, emulsion stability, and the size of dispersed phase. They have reported lower temperature rise with an increase in emulsification time, evenly distributed and smaller size of dispersed phase, and highest stability with oil-in-water emulsion [58].

With the fact that the three-phase oil-in-water-in-oil emulsion uses two-stage emulsification processes and requiring two types of surfactants, generally the process cost would be greater than that of a two-phase emulsion process. Although the above mentioned literature documented the advantages of three-phase emulsions over the two-phase emulsions, it is not yet clear where the boundary lies as far as the process cost, emulsion characteristics, emulsified fuel properties, engine performance, and emission are concerned. It is also not clear about the comparison of the microexplosion process of two-phase and three-phase emulsion. A further study on the microexplosion phenomenon of three-phase oil-in-water-in-oil emulsion fuel inside a combustion chamber atmosphere is recommended.

### 3.2. Two-Phase Emulsion

**Figure 2:**
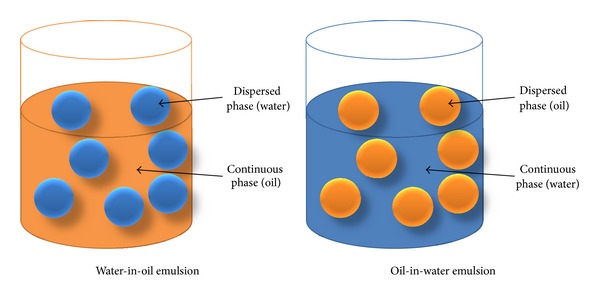
Concept of two-phase water-in-oil and oil-in- water emulsions.

There are two basic forms of two-phase emulsion. The first is the oil-in-water (O/W) emulsion in which oil droplets are dispersed and encapsulated within the water column. The second is the water-in-oil (W/O) emulsion in which droplets of water are dispersed and encapsulated within the oil.  Figure 2 shows the concept of two-phase water-in-oil and oil-in-water emulsions. For either type of stable emulsion to form, three basic conditions must be met [62].

(a) The two liquids must be immiscible or mutually insoluble in each other. (b) Sufficient agitation must be applied to disperse one liquid into the other. (c) An emulsifying agent (surfactant) or a combination of emulsifiers must be present. In addition to the above emulsion, few researches included concepts of introducing three-phase emulsions and comparative studies on the effect of two-phase and three-phase on the diesel engine performance also available. Irrespective of method of production either by mechanical homogenizing or by ultrasonic vibrating, the oil-in-water-in-oil (O/W/O) emulsions were found to have a higher fuel consumption rate, brake specific fuel consumption, CO emission, and black smoke opacity than the W/O emulsions [30, 31]. Surfactants used for the formation of water-in-diesel emulsion fuel should burn easily with no soot and should be free of sulphur and nitrogen as discussed in [5]. Furthermore, they should have no impact on the physicochemical properties of the fuel. Surfactants from the aliphatic hydrocarbon family are the best candidates to be used as emulsifiers. Usually the amount of surfactants used for emulsification is in the range of 0.5–5% by volume, as the surfactant concentration increased emulsion stability reduced.

## 4. Effect of Emulsification on Stability and Physiochemical Properties of WiDE

The stability of the diesel emulsion is affected mainly by the emulsification technique, emulsification duration, volume fraction of water (dispersed phase), viscosity of continuous phase (diesel oil), stirring speed (or ultrasonic frequency), and concentration of surfactants. The experimental work by Chen and Tao [62] studied the effect of emulsifier dosage, oil water ratio, stirring speed, and emulsifying temperature on the stability of water in diesel emulsion using mechanical agitator. They reported that an increase in oil to water ratio, stirring speed, and duration had positive influence on stability, whereas an increase in emulsifying temperature showed negative impact. Two-phase W/O emulsion showed better engine performance with less CO emissions which were reported in [30] with the application of ultrasonic vibrator compared to the emulsion prepared by mechanical agitation. In addition, the selections of suitable surfactants, the choice of a suitable agitator frequency, and agitation time have also been identified as equally important parameters in the formation of stable emulsified fuels [63].

Surfactant or emulsifier is the most important factor that affects the stability of an emulsion. Percentage of water in the emulsion, stirring intensity, stirring duration, emulsifying temperature, and operational pressure also affect the stability of an emulsion. Chen and Tao [62] have experimentally studied the effect of emulsifier dosage, oil-water ratio, stirring speed, and time and emulsifying temperature on the stability of diesel-water emulsion. They have concluded that an emulsifier dosage of 0.5%, oil-water ratio of 1 : 1 by volume, stirring speed of 2500 rpm, and duration of 15 min and emulsifying temperature of 30°C have been optimum for the stability of the emulsion. They have also reported that while an increase in oil to water ratio, steering speed, and duration up to 15 min have positive impact on stability, increase in emulsifying temperature had negative impact. Similar work has been done by Ghannam and Selim on the stability of water/diesel emulsion fuel and they indicated the necessity of surfactant for the stability of the emulsion and possibility of getting stable emulsion of higher water percentage (>30%) by increasing the percentage of emulsifier agent (2%) and increasing speed up to 20000 rpm with mixing period of 30 min [64].

To be a good fuel for a compression ignition engine, a water-in-diesel emulsion should possess most of the positive effects of petrodiesel fuel. As this type of engine is well established, complete alteration of fuel characteristics that requires engine retrofitting would not be feasible economically. A good CI engine fuel should hold characteristic features like short ignition lag, sufficiently high cetane rating in order to avoid knocking, suitably volatile in the operating range temperatures for good mixing and combustion, easy startup characteristics, limited smoke and odor, suitable viscosity for the fueling system, free from corrosion and wear, and ease of handling [65]. In diesel engines, fueling system must insure the fuel to be delivered into the engine cylinder economically and in an appropriate time so that it runs smoothly with minimal exhaust and noise. This is done by controlling the process of spray penetration, chemical and physical atomization, mixture ignition, and combustion and exhaust gas formation. These are mainly dependent on the physiochemical behaviors of the fuel and the injection system. Significant number of the literature could be found on the physiochemical behavior of water-in-diesel emulsion as well as their effect on the combustion behavior and stability. As the water content of the emulsion increases, physical properties like density [7, 32, 66], viscosity [7, 26], bulk modulus of elasticity [7], and compressibility [44] increased. A very close attention should have to be given to these changes as density has a pronounced effect on the mixing process and viscosity in the injection system. It has also been reported that water addition reduces the heating value of the emulsion.

## 5. Effect of WiDE on Combustion Process

In a WiDE, water remains embedded inside the diesel droplets with the help of the surfactants. When this type of emulsion is sprayed into a hot combustion chamber, heat is transferred to the surface of the fuel droplets by convection and radiation. Since water and diesel have different boiling temperatures, the evaporation rates of these two liquids will be different. As a result, the water molecules reach their superheated stage faster than the diesel, creating vapor expansions breakup [17, 20, 37, 40, 58, 67]. It is at this stage that the two phenomena, microexplosion and puffing, prevail. Microexplosion is that the whole droplet breaks up into small droplets quickly, while in puffing, water leaves the droplets in a very fine mist (a part of the droplet break up) [22, 26].

**Figure 3:**
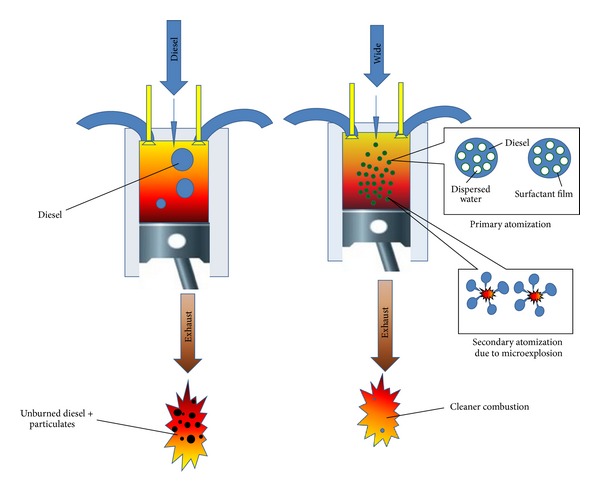
Primary and secondary atomisation in spray flame of emulsified fuel.

These microexplosions result in a fast breakdown, or secondary atomisation, of the fuel droplets which in turn causes fast fuel evaporation and hence an improved air-fuel mixing as illustrated in Figure 3. Therefore, it is equally important to study the basics of microexplosion of water-in-diesel emulsion and its influencing parameters as it plays a major role in the combustion improvement.

According to Morozumi and Saito, microexplosion is mainly affected by the volatility of the base fuel, type of emulsion, and water content. Based on their conclusion, an increase in an emulsifier content increases the microexplosion temperature and waiting time [20]. Mechanisms of microexplosion and their dependence on various parameters affecting microexplosions have been extensively investigated by Fu and coworkers [40]. They have stated that both water-in-oil and oil-in-water emulsions can microexplode at certain conditions. Furthermore, they have related the diameter of the dispersed liquid with the strength of the microexplosion through a physical model.

The advantages on performance and emissions of water-in-diesel fuel and factors affecting microexplosion have been extensively researched. Fu [68] and coworkers have challenged its occurrence inside diesel engine combustion chamber. Based on their conclusion, the droplet diameter of an emulsion in the combustion chamber is in the range of 20–30 *μ*m and microexplosion phenomenon could not occur with this range of droplet sizes [38]. Even though this report is agreeable with the reports that state the effect of mean water particle size diameter on the intensity of microexplosion [39, 69], it contradicts with most of the literature on the occurrence of microexplosion in diesel engine combustion chamber.

Microexplosion is an important phenomenon in the secondary atomization process of water-in-diesel emulsion fuels. Generally, this phenomenon is affected by volatility of base fuel, type of emulsion, water content, diameter of the dispersed liquid, location of the dispersed liquid, and ambient conditions like pressure and temperature. Although many studies have been conducted both experimentally and numerically to understand the phenomenon of microexplosion, yet the study of its effects inside the combustion chamber are quite few. It is believed that fuel injection and the passage of emulsified fuel through the narrow exit of the injection nozzle affect the dispersed liquid behaviour of the fuel. It is therefore very important to study the microexplosion phenomenon inside a combustion chamber and its effect on the combustion process like the secondary atomization, spray penetration, evaporation, and mixture ignition.

Combustion process is generally characterized by factors such as injection characteristics, spray penetration, evaporation, chemical and physical atomization and mixture ignition, engine cylinder pressure and temperature, and heat release characteristics [8, 22, 41, 70]. As far as the fuel-injection characteristics is concerned, it is observed that the injection pressure profile extension over a longer period leads to retarded injection timing and 22–26% increase in injection duration [41]. Armas et al. have also reported similar results on injection and they have associated it with an increase in viscosity of the emulsified fuel [7]. Ochoterena et al. have studied the spray behaviors of WiDE, water-in-diesel microemulsion and conventional diesel on high pressure and high temperature constant volume chamber, keeping an eye on the penetration and lift-off, cone angle measurements, start of combustion measurements, and singularities of atomization. A longer droplet penetration and wider cone angle with the emulsified fuel compared to pure diesel fuel was observed which was associated with a lower volatility of water [22]. A slightly longer ignition delay, same report by Ghojel and Honnery [41] and Armas et al. [7], and longer combustion duration were also reported with the emulsion fuel, both as a result of lower flame temperature. Ignition delay up to 29% was reported when WiDE as fuel in an HSDI diesel engine [45]. In another experiment Subramanian et al. [46] reports that the ignition delay is much higher with WiDE as compared to water injection to the manifold during the intake stroke. The effect of water content, the injection pressure, and ambient temperature on ignition delay was further studied by Ghojel and Tran [27]. Ambient temperature significantly affected the ignition delay. On the other hand, no significant effect was observed with injection pressure. They have also studied the effect of water content, the injection pressure, and ambient temperature on flame lift-off. They have reported an increase in flame lift-off with an increase in injection pressure and water content while it decreased with an increase in ambient temperature. Alam Fahd et al. [11] has experimentally found that pressure traces and heat release rate were comparable with respect to pure diesel at different speed and loading conditions.

## 6. Effect of WiDE on Engine Performance

The effect of volume percentage of water added in the emulsion on the performance of an engine was studied by many researchers [26, 32, 33, 44, 47–50, 70]. Abu-Zaid has studied torque, power, brake specific fuel consumption, and brake thermal efficiency by varying the volume percentage of water from 0 to 20% water/diesel ratio with 5% resolution [47]. Alahmer et al. studied the above mentioned engine performance parameters in a four stroke, four-cylinder direct injection engine by varying the volume percentage of water from 0 to 30% water/diesel ratio with 5% resolution [33]. The experimental investigation by Selim and Elfeky [70] on the other hand has used 0, 2, 4, 6, and 8% by volume of water in the emulsion to study its effect on the heat flux on the engine components. Water contents of 5%, 10%, and 15% by volume were used in the study of their effect on the engine performance parameters (torque, power, brake mean effective pressure, and specific fuel consumption) [32]. Here, surfactant type was also taken as a variant to see the effect of gemini surfactant on the engine performance parameters and its comparison with conventional ones. Park et al. have experimentally studied the effect of the volume percentage of water on the combustion characteristics of an emulsion fuel in a rapid compression and expansion machine by considering 0, 16.67%, and 28.6% of water by volume in the emulsion [26]. In another study, Park et al. have experimentally investigated the combustion characteristics and engine durability of a four-stroke, six cylinder direct injection diesel engine with a turbocharger used as a power unit in city/highway bus fueled by pure diesel, 13%, 15%, and 17% of water by volume in the emulsion [48]. Kannan and Udayakumar have also experimentally studied the effect of water percentage of water emulsified diesel fuel on the brake thermal efficiency, brake specific fuel consumption, NO_*x*_, and hydrocarbon emissions in a single cylinder four stroke direct injection diesel engine by considering 0, 10%, and 20% of water by volume in the emulsion [44]. On a separate study by Samec et al., water contents of 0, 10%, and 15% by volume were considered for the experimental investigation of the effects of water content on the combustion characteristics of diesel engine [49, 50]. This experimental work was accompanied with a numerical investigation.

As far as the combustion performance and emission (refer to Table 1) of diesel engines fueled with WiDE are concerned, inconsistent results have been reported by different researchers. Besides, all the reports are based on different engine setups and methodologies. As a result, an optimum percentage of water content in the emulsion cannot be drawn. But we can conclude from this that water content ranging from 5 to 40% by volume in the emulsion can be utilized for fueling transport and stationary diesel engines. A systematic approach of studying the optimization of water content in the emulsion for best engine performance and emission by both experimental and numerical investigations is necessary that can give best recommendation for the commercialization of WiDE as an alternative source of energy of the future diesel engines.

### 6.1. Engine Torque

Abu-Zaid, on his study of the effect of water content on the engine performance, has reported that the engine torque increases with an increase in the percentage of water in the emulsion [47]. According to Alahmer et al. [33], maximum torque was reported when the engine is fueled with a 5% water content by volume emulsified fuel. A reduction in torque with an emulsified fuel compared to the pure diesel fuel is reported by Nadeem et al. A relatively comparative torque is registered with 5% water by volume in the emulsion and gemini surfactant as an emulsifier used. The cause for the torque reduction in the emulsion is due to the reduction in heating value with an addition of water [32].

### 6.2. Engine Power

Abu-Zaid, on his study of the effect of water content on the engine performance, has reported that the engine power increases with an increase in the percentage of water in the emulsion [47] while Alahmer et al. reported that maximum power was achieved when the engine was fueled with a 5% water content by volume emulsified fuel [33]. On the other hand, Nadeem et al. have reported infinitesimal difference with the power output of the engine in the speed range of less than 4000 rpm. Even at 4000 rpm, pure diesel exhibited better power output compared to all emulsified fuels, with a relatively nearer performance with emulsified fuels using gemini surfactant [32]. A power loss of 7-8% was also reported by Barnes et al. on their application of WiDE with 10% water content by volume [71]. Since these results are based on different engine setups and methodologies, it is very difficult to explain the conflicting results reported on the engine power.

### 6.3. Engine Brake Specific Fuel Consumption

Brake specific fuel consumption (BSFC) was studied by Abu-Zaid by considering two cases. The first analysis has considered the total fuel as a sum of both the quantity of diesel and water resulting to an increased BSFC with an increase in the percentage of water in the emulsion. The second considered diesel alone as a total fuel and the analysis resulted with a decrease in BSFC with an increase in the percentage of water in the emulsion, the minimum value is reported to be at 20% water in the emulsion. The main reason for the reduction in BSFC is due to the secondary atomization of spray because of microexplosion [47]. This result was also shared by another publication by Kannan and Udayakumar on their experimental study on the effect of water percentage of water emulsified diesel fuel on BSFC. They have found that the BSFC of the engine decreases with an increase in the volume percentage of water in the emulsion, minimum value reported when the volume percentage of water was at 20%. This attribute, based on the report, is due to the displacement of diesel by water, resulting in less amount of diesel contained in the emulsion [44]. On the other hand, on separate study by Ghojel et al., 22–26% increase of BSFC was reported with emulsified diesel fuel of 13% water content by volume compared to diesel fuel [8]. Alahmer et al. have classified the effect of water percentage on the BSFC at high speed and low speed. According to their report, there has been an increase in BSFC with an increase in the percentage of water in the emulsion when the engine was at higher speed. There was no significant effect reported on BSFC with an increase in the percentage of water in the emulsion when the engine was at lower speed. Lowest engine BSFC was also reported with pure diesel fuel compared to emulsified fuels, with 15% water content emulsion taking the highest value [33]. The main factor attributing to this situation according to the authors is due to the displacement of diesel fuel with the amount of water added, which further will facilitate the fuel burning in the precombustion. An increase of BSFC in the range of 2–7% was reported by Barnes et al. on their study of effect of water blended fuel on the performance and emissions of a city bus engine considering a 10% water content by volume [71]. Armas et al. has also reported an increase in brake specific fuel consumption with 10% water content by volume in the emulsified fuel compared to pure diesel fuel [7].

While Abu-Zaid [47] and Kannan and Udayakumar [44] both reported improvement in BSFC with an increase in the percentage of water content in the emulsion, negative effect on BSFC was also reported [8, 32, 33, 71]. Higher BFSC irrespective of engine loading was reported by Alam Fahd et al. [11].

### 6.4. Engine Brake Thermal Efficiency

Kannan and Udayakumar have experimentally studied the effect of water percentage of water emulsified diesel fuel on brake thermal efficiency. They have found that the brake thermal efficiency of the engine increases with an increase in the volume percentage of water in the emulsion. This attribute as reported by the authors is due to an increase in expansion work and reduction in compression works as a result of expansion of water vapors [44]. Slight improvement in thermal efficiency was also reported by Armas et al. and Ghojel et al., 3.5% increase in brake thermal efficiency was reported for the study of engine fueled with 20% water in the emulsion according to Abu-Zaid [47]. Alahmer et al. reported that maximum brake thermal efficiency was achieved when the engine is fueled with a 5% water content by volume emulsified fuel [33]. with a static injection timing of 23° BTDC; Subramanian [46] compared the effects of WiDE and direct injection of water into the manifold and found WiDE to be more effective than injection of water with regard to brake thermal efficiency.

## 7. Effect of WiDE on Emissions

The introduction of water by the emulsification process has many effects on the combustion process that have direct consequences on the pollutant formation. Vaporization of water due to heat absorption from its surroundings will lower the local high temperature resulting in the reduction of NO_*x*_ [5–8, 21, 26, 33, 44]. Alahmer et al. on their study of water emulsion on the performance and emission have reported that at low amount of water addition, the amount of emitted NO and NO_*x*_ increases, but at high water content, the amount of emitted NO and NO_*x*_ decreases [33]. Furthermore, Kannan and Udayakumar have mathematically modeled nitric oxide formation in single cylinder direct injection diesel engine using diesel-water Emulsion [72]. Based on their results, it was found that 18% and 21.5% of reduction in NO was achieved with 10% and 20% dilution of diesel with water, respectively. On their experimental investigation in another literature, the same authors have reported that 10% and 25% reduction of NO_*x*_ in a single cylinder diesel engine for 10% and 20% water in the emulsion was observed, respectively [44]. Ghojel and coworkers have reported 29–37% reduction of NO_*x*_ emissions when operating on diesel oil emulsion of 13% water content by volume [8]. Another experimental and numerical study conducted by Samec et al. reported a reduction of 20% and 18% NO_*x*_ emission compared to pure diesel fuel with 10% and 15% water content in the emulsion, respectively [49, 50]. A decrease of NO_*x*_ emission of 9% was reported by Barnes et al. on their study of effect of water blended fuel on the performance and emissions of a city bus engine considering a 10% water content by volume [71].

Likewise, there is also a microexplosion phenomenon as it has been discussed in detail. The effect of microexplosion is to facilitate the mixing process, in turn, it will reduce reaction time. Furthermore, the reduction in maximum local temperature also reduces the reaction rate. These combined effects reduce the formation of particulate matter and soot [7, 21] and total hydrocarbon [7, 8, 44] in the exhaust. The HC is also further reduced with the effect of OH radical that is dissociated from water [7, 44]. Ghojel et al. on their study of performance, emission, and heat release characteristics of direct injection diesel engine using diesel oil emulsion, have reported 60–90% reductions of HC emissions when operating on diesel oil emulsion of 13% water contents by volume compared to the base fuel [8]. Samec et al. reported a reduction of 52% and 33% total hydrocarbon emissions; and 68% and 75% reduction of soot emission compared to pure diesel fuel with 10% and 15% water content in the emulsion, respectively [49, 50]. Barnes et al. have reported a 20% decrease in PM emission on their study of effect of water blended fuel on the performance and emission of a city bus engine considering a 10% water content by volume [71]. Reduction in exhaust temperature and less CO has been reported for all engine loading conditions [11] but higher CO at low load, low speed was significantly reduced at higher engine rpm. At low load conditions of HSDI engine for 25.6% water to fuel ratio, NO_*x*_ is most often reduced upto 50% with 94% reduction in PM [45]. On the whole, WiDE is more effective in reducing NO and smoke level at low engine loads [46]. On the other hand, it is reported that there is an increase of CO_2_ [33] and CO [5] emissions with water-in-diesel emulsion compared to the base diesel fuel. This is because of excess oxygen in the combustion mixture.

Armas et al. investigated the effect of 10% water addition with diesel on the emission levels of NO_*x*_, total hydrocarbons (THC), soot, particulate matter (PM), and its composition [7]. There is a relative reduction in most of the pollutant emissions when the engine is operated with 10% water-in-diesel emulsion agreed with most of the literature in this area. According to a report by Sadler [73], an application of 13% water content (not mentioned whether by volume or mass percentage) in the emulsified fuel in UK has brought 13% and 25% reduction of NO_*x*_ and PM, respectively.

According to a report by Nadeem et al., [32] on their study performance and emission using conventional and gemini surfactant stabilized emulsified fuels, lowest PM, NO_*x*_, and CO were produced by the engine when it was operated using emulsified fuel containing 15% water contents with gemini surfactants. Lin and Wang [57] on their study of engine performance and emissions characteristics using a three-phase emulsion prepared by two-stage emulsification method have reported an increase in CO_2_ and CO emissions and decrease in O_2_ and NO_*x*_ emissions with the emulsified fuel compared to neat diesel fuel. On their comparison between two-phase and three phase emulsions, three phase emulsion fuel has registered lower CO and NO_*x*_ emissions. A similar experimental study has been conducted by Lin and Chen [30] on a four cylinder diesel engine to compare fuel property and emission characteristics of two-phase and three-phase emulsions prepared by ultrasonic vibrator and mechanical homogenizer. They have reported results for NO, CO, CO_2_, O_2_, and smoke opacity. Largest content of NO was emitted when the engine was fueled with neat diesel fuel while three phase emulsion fuel prepared by mechanical homogenizer had lowest NO emission. With regard to CO emission, lower emission was registered with a two-phase emulsion fuel prepared by ultrasonic vibrator. A similar trend has been observed by CO_2_ and O_2_ emissions with all fuel types. Highest smoke opacity was registered with neat diesel fuel while lowest emission was observed with three phase emulsion fuel prepared by mechanical homogenizer.

## 8. Conclusions and Future Recommendation

WiDE fuel has become the best alternative fuel to substitute diesel fuel in both transport and stationary CI engines. The driving force for the growing interest to this type of fuel is simultaneous reduction of both NO_*x*_ and particulate matters. This occurs as a result of the reduction in peak cylinder temperature and secondary atomization by a further breakup of fuel spray due to microexplosion. Although score of research have been conducted both experimentally and numerically outside the engine, studies of its effects inside the combustion chamber were quite few. Experimental investigation about the effect of various surfactants in the WiDE on engine performance and pollutant formation is not known. This review paper emphasise the research gap to investigate the effects of various surfactants with several blends of emulsified fuel on the combustion characteristics, emission formation processes, and engine behaviours also to determine the pollution formation suppression capability of the emulsified fuels by in-depth combustion characteristics analysis.

It is also equally important to select the suitable emulsification technique, optimised speed and agitation time in order to achieve stable emulsion.

There have been inconsistent results reported by different researchers with regard to the effect of water content on the engine combustion characteristics. Besides, all the reports are based on different engine setups and methodologies. As a result, an optimum percentage of water content in the emulsion cannot be drawn. But it can be concluded that water content ranging from 5–40% by volume in the emulsion has been utilized in the experimental and numerical investigation.

There was a common agreement by most of the researchers on the report of the effect of water content on the simultaneous reduction of both NO_*x*_ and particulate matter. The inconsistency was on the percentage amount reduction compared to pure diesel. Up to 37% reduction NO_*x*_ and 90% reduction in particulate matter were reported by different researchers. A systematic approach of studying the optimization of water content in the emulsion for best engine performance and emission by both experimental and numerical investigations is necessary so that it can give the best recommendations for the commercialization of WiDE as an alternative source of energy for the future diesel engines.

## Conflict of Interests

The authors declare that there is no conflict of interests regarding the publication of this paper.

## Figures and Tables

**Table 1:** Engine Performance for WiDE under various testing conditions.

Reference	Engine type and loading conditions	% of water	Surfactant used	Amount of surfactant used	% increase in specific fuel consumption	% increase of torque	% increase of Brake thermal efficiency	% reduction NO_*x*_	% reduction of PM	% reduction of HC and CO
[7]	Renault F8Q turbo charged intercooler IDI,5 different steady state operating conditions	10	Polyethylenglycole monooleate and sorbitol sesquioleate	NA			Reduced	Reduced	Reduced	HC reduced
[8]	4s, 4c, di industrial diesel engine	NA	NA	NA	22–26% compared with certified diesel fuel (CDF)	NA	Slightly Higher than CDF	29–37% reduced	Not measured	60–90% reduced HC
[11]	2.5 L DI turbo-charge Toyota diesel engine, 25%, 50%, 75%, and 100% load with 800–3600 rpm in steps of 400 rpm	10% water	10% biodegradable surfactant	10% by volume	Increased in all test conditions	NA	Increased with speed	Reduced	NA	Higher at low load and decreasing with increasing speed and load
[32]	FORD XLD 418, 1000–5000 rpm	5, 10 and 15	Conventional-sorbitan monooleate (SM) and gemini surfactant	0.5% for SM 0.4% for gemini	15% water has highest and decreases with decrease in water content	Less with all emulsions compared to diesel 5% water produced highest torque	NA	Reduced	Reduced	Lowest with 15% water
[33]	4 cylinder, DI water cooled1450cc, 1000–3000 RPM	5–30% insteps of 5	Polysorbate-20	2% by volume	Increased	At 5% water torque was max., and declining with increase of water content	For 5% water = 35%	NO and NO_*x*_ reduced with increase in water	Reduced	HC and CO_2_ increased with increases in water content
[41]	4C,4S, water cooled DI industrial HINO diesel engine, 200 Nm and 2200 rpm	13	NA	2% (surfactants and cetane improver)	Increase of 26%	NA	NA	NA	NA	NA
[44]	Single cylinder, 4S, DI diesel engine with injection pressure of 200 bar, constant speed 1500 rpm	10% and 20%	Sodium laurel sulphate	0.1% for 1000 mL emulsion	Break BFC decreases with all load	NA	Increase with increased water content	Reduced 10% for 10% water and 25% for 20% water	NA	Decreasing with all loading conditions
[45]	4 cylinder, HSDI diesel engine at 1480, 2035, 1480, 2065, and 1460 rpm	20	Span 80 and Tween 85	1.3% of Span 80 and 0.7% of Tween 85	BSFC increased with increased EGR rate	NA	NA	Reduced between 30–50% at low injection pressure and increased up to 24% at higher injection pressure	94% reduced at low loads	
[46]	4S, air cooled overhead valve, constant speed of 1500 rpm at different outputs.	0.4 : 1 ratio	Surfactant used unknown, with HLB 7	NA	NA	NA	NA	NO_*x*_ is reduced	NA	NA
[47]	Single cylinder DI diesel engine, 1200–3300 rpm	0–20% in steps of 5%	Span 80 and Tween 80	2% by volume of mixture	Decreased with increasing water content	NA	Appr-3.5% for 20% water	NA	NA	NA
[48]	6 cylinder, TCI diesel engine (High way Bus Engine), 10, 25, 50, 75 and 100% of full power at 1200 rpm and 2000 rpm	15% analysis considered for 15% water content	NA (used along with cetane improver)	NA	at 25% and 50% load slightly higher than diesel. At 75% BSFC is better than diesel	Decreased 20% and 9% at 1200 rpm and 2000 rpm when compared with diesel	NA	Reduced up to 11.6%	Reduced up to 34.5%	CO and HC increased up to 12.4% and 59.4% respectively
[49]	4 cylinder air-cooled DI truck diesel engine	0, 10, 15	(Span 85) Quantity NA	NA	NA	NA	NA	20 for 10% water 18 for 15% water	NA	THC reduced about 52% for 10% water 33% for 15% water
[50]	4 cylinder, air cooled, 1700 rpm and 2100 rpm	10 and 15%	NA	NA	NA	NA	NA	Reduction of 20% and 18% for 10% water and 15% water	NA	THC reduced 52% and 33% for 10% water and 15% water
[51]	6 cylinder, Caterpillar 3176 turbocharged engine, steady state operation	20% by mass	Purinox (commercial DE fuel)	NA	0.7% reduced	NA	NA	19% reduced	16% reduced	HC and CO emissions increased by 28% and 42%
[52]	Renault VI 620–45 (Euro 1) engine testing	13% by weight	NA	2-3%	Reduced 1–4%	NA	NA	Appr. 30% with reduction of 80% black smoke	Up to 50%	12% reduced HC
[53]	2.5L, 4cylinder.D.I. Ford engine, different load with 2500 rpm	20% by vol.	NA	NA	NA	NA	NA	Decrease Up to 60% with increased smoke	NA	HC and CO increased relatively low level
